# The ‘Bounce Back’ rehabilitation programme for stroke survivors in South Africa – a case report

**DOI:** 10.4102/sajp.v81i1.2117

**Published:** 2025-02-21

**Authors:** Laeeqa Sujee, Sacha Hildebrandt, Amy Harrison, Christa Matjekane, Thabiso Mmoledi, Sonti Pilusa, Kganetso Sekome, Adedayo T. Ajidahun

**Affiliations:** 1 Department of Physiotherapy, Faculty of Health Sciences, University of the Witwatersrand, Johannesburg, South Africa; 2 Phila Sonke Wellness Initiative, Johannesburg, South Africa

**Keywords:** stroke, goal setting, return to work, quality of life, participation, mental health

## Abstract

**Introduction:**

Stroke is one of the global leading causes of disability, with a higher prevalence at a younger age in sub-Saharan Africa. Returning to functional status is a primary goal of rehabilitation for stroke survivors. However, the cost of intensive rehabilitation is often a barrier for the under-resourced in South Africa.

**Patient presentation:**

This article describes the individualised ‘return-to-function’ approach to rehabilitation and its outcomes for five stroke survivors delivered through a non-profit public–private partnership in a low-income community in South Africa.

**Management and outcome:**

A retrospective case series of stroke survivors who participated in the Bounce Back Journey (BBJ) programme between 2019 and 2021 was conducted. The beneficiaries’ ages ranged from 21 to 55 years; two were females and three were males. The goals and outcomes of five stroke survivors with impairments, functional limitations and participation restrictions admitted into the BBJ programme were evaluated through standardised outcome measures. All the beneficiaries required financial support and extensive rehabilitation to return to a functional life. All showed improved outcomes in functional independence and health-related quality of life at discharge. Depressive symptoms clinically deteriorated in one patient. At discharge, all participants faced challenges finding opportunities to return to work.

**Conclusion:**

The case series demonstrates how an alternative community-based rehabilitation programme has the potential to improve functionality, health-related quality of life and mental health.

**Contribution:**

A low-cost, community-based, intensive rehabilitation programme can improve functionality, but return to work and community re-integration opportunities remain limited.

## Introduction

Stroke is the third most common cause of adult physical disability and the second greatest cause of mortality in the world, accounting for more than 6 million deaths annually (Feigin et al. 2021). Most stroke-related deaths take place in low- and middle-income countries (LMICs) (Maredza, Bertram & Tollman 2015). Experiencing a stroke comes with multiple long-term health challenges that can worsen the primary disability.

Disability is a biopsychosocial phenomenon, depicted by the International Classification of Functioning, Disability and Health (ICF) model which describes disability as the interaction between impairment of body functions, structures, activities and participation in the context of the person’s environmental and personal factors (World Health Organization 2002). Functional outcomes and activities of daily living (ADLs) are affected in two-thirds of stroke survivors (Rosamond et al. 2007). Some of the common impairments to body functions and structures limitations presenting post-stroke are upper and lower extremity paralysis or paresis, mental health challenges and speech problems (Silva et al. 2015). The ICF has been used to evaluate disability in people post-stroke (Perin et al. 2020).

Collaborative care comprising interdisciplinary teams is recommended in stroke rehabilitation (Alessandro et al. 2020). The Phila Sonke Wellness Initiative (PSWI) Bounce Back Journey (BBJ) rehabilitation programme, based on donor funding, is an evidence-based programme developed on the person-centred social model. It uses an interdisciplinary approach to restore functional independence (FIM) and social integration and identify the best fit for means of livelihood and income for participants to improve their overall quality of life (O’Dell 2016).

This case series presents the outcomes of the BBJ programme which is a community-based, integrated team approach to stroke survivors’ rehabilitation and return to an independent functional state.

## Management

### Stroke survivor selection

Participants were stroke survivors living in Dobsonville, South Africa. The stroke survivors were referred to PSWI via doctors and rehabilitation healthcare providers in the public sector as well as through word of mouth. The stroke survivor beneficiary manager who is an occupational therapist screened each potential stroke survivor to be included, in two phases. The first phase involved a non-clinical screening, during which the application and all documentation were reviewed. The inclusion criteria were individuals older than 13 years, unable to receive intensive rehabilitation in the public or private sector because of resource scarcity, long waiting lists and a lack of capacity to see patients more frequently. Participants were excluded if the stroke incident was more than 18 months prior to application, if they were unwilling to share accurate financial information about their household income, if their primary disability was mental or psychological as per medical doctor diagnosis and if there had been a recent history of drug and substance abuse. There were no other limitations based on the number of previous strokes or comorbidities.

### Intervention programme

**FIGURE 1:**
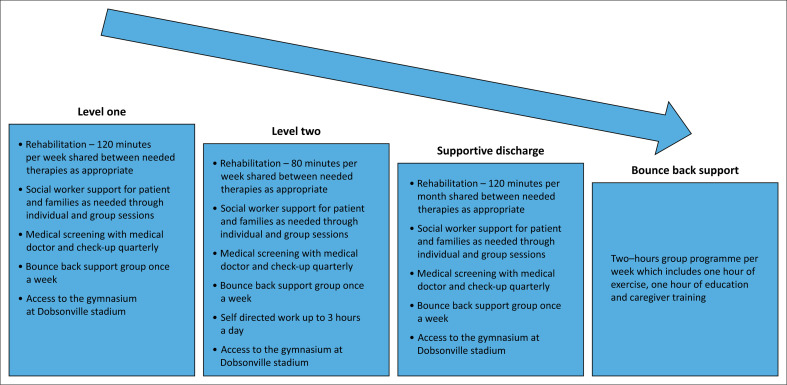
Bounce back journey rehabilitation programme.

The second phase involved the BBJ rehabilitation programme which was a graded intervention programme delivered by healthcare professionals with a special interest in neurorehabilitation. The BBJ rehabilitation programme is based on the person-centred care framework that includes the person’s well-being, context, expression, preferences and beliefs (Ekman et al. 2011). The programme involved the stroke survivor starting on a specific level based on the initial assessment, and then the intensity of therapy was weaned down as goals were achieved. This slow grading strategy aimed to support the stroke survivors and encouraged their empowerment and independence. Stroke survivors were allocated to a certain therapy intensity level approved by a case management team comprised of occupational therapists and physiotherapists, for a particular period of rehabilitation based on the assessments of the stroke survivor beneficiary manager and the recommendations of the rehabilitation team. The rehabilitation team included a physiotherapist, occupational therapist, speech therapist, social worker and medical doctor. The programme was implemented at no cost to the stroke survivor and was fully funded through the non-profit organisation PSWI. The length of stay in the levels was dependent on the patients’ outcome and level of independence. At supportive discharge, all the participants had been in the programme for at least 9 months. The content of the programme is outlined in Figure 1.

### Outcome measures

#### Functional independence

The FIM+FAM measure is a 30-item scale that measures dependence. The domains measured include – self-care, transfer and mobility, communication, cognition and psychosocial function (Turner-Stokes et al. 1999). The tool is validated with stroke survivors and has high internal consistency (Cronbach alpha = 0.98) (Turner-Stokes & Siegert 2013). Higher scores on the FIM+FAM measure show increased independence; the lower the score, the more dependent the patient is (UK FIM+FAM Users Group 2012). Formal permission for use is not required.

#### Depression

The Center for Epidemiologic Studies Short Depression Scale (CES-D10) is a 10-item questionnaire developed to measure depression symptoms among adults. The tool has been validated among the South African general population (Baron, Davies & Lund 2017) and shows high internal consistency (Cronbach’s α = 0.86). The higher the score, the higher the depressive symptoms, with a score of ≥ 10 showing substantial symptoms of depression (Andresen et al. 1994).

#### Health-related quality of life

The EQ-5D-5L questionnaire measures health-related quality of life. The tool has been validated in several patient groups, including patients with stroke. The EQ-5D-5L is a valid and reliable tool that measures health changes. Formal permission for digital use was obtained from the EuroQol Research Foundation. The lower the health-related quality of life scores, the fewer problems the patient faces (Oeman & Jansen 2013).

### Procedure

There were two teams involved in the programme. The first team was the case management team which comprised rehabilitation professionals. It included a stroke survivor beneficiary manager, programme coordinator, research coordinator and a moderator who facilitated review meetings. The stroke survivor beneficiary manager contacted the potential stroke survivor if they met the initial inclusion and exclusion criteria based on their application, and they were then invited for an in-person clinical assessment. The assessment included baseline measurements with three outcome measures: quality of life (EQ-5D-5L), depression (CES-D10) and FIM/FAM. This assessment was to confirm the potential stroke survivor’s capabilities based on the outcome measures. In addition, the stroke survivor beneficiary manager assessed body impairments, activity limitations and participation restrictions as per the ICF framework. This assisted the case management team in making the decision regarding which level of the BBJ programme the stroke survivor should be included in. Once the case management team had allocated the stroke survivors to an appropriate programme level, they were enrolled and referred to the second team involved in the programme, the rehabilitation team which carried out interdisciplinary assessments. These included a first medical appointment, a physiotherapy assessment, an occupational therapy assessment, a speech therapy assessment and a social work assessment. These assessments were person-centred and at the discretion of each therapist. The assessments allowed the therapy team to allocate adequate time and schedule rehabilitation sessions for the stroke survivor as required.

It was paramount that the stroke survivor set SMART (specific, measurable, attainable, realistic and time-bound) goals during these initial sessions so that a plan regarding therapy could be determined between the therapist and the stroke survivor. All goal setting was carried out as a treatment strategy, where therapists spent time with the stroke survivor to help them formulate goals in their own words. This emphasised the stroke survivor-centred, stroke survivor-driven and therapist-guided approach that the initiative strove to achieve.

The stroke survivor beneficiary manager held monthly meetings with the rehabilitation team to discuss each stroke survivor’s goals and progress as well as brainstorm different treatment and management techniques and approaches. Quarterly, the stroke survivor beneficiary manager met with each stroke survivor to reassess the outcome measures of FIM, depressive symptoms and health-related quality of life. These sessions also included discussions with the stroke survivor regarding their progress, their needs and what they hoped to achieve in rehabilitation going forward. The results were then reviewed by the dedicated case management team alongside the beneficiaries’ goals, outcomes and recommendations from the rehabilitation team. These findings allowed the case management team to determine a change in programme level for the next quarter as well as address requests or concerns from the stroke survivor and rehabilitation team.

### Ethical considerations

Ethical clearance to conduct this study was obtained from the University of the Witwatersrand, Human Research Ethics Committee (reference no.: M231090). Written informed consent was provided by all the beneficiaries.

## Patient presentation and outcomes

### Sample description

Five stroke survivors who had a stroke between 2019 and 2021 were managed under this programme. The stroke survivors’ median age was 39 (IQR:27.5-48) years. There were two females and three males (Table 1).

**TABLE 1:** Description of the stroke survivors.

Stroke survivor	Age at stroke (years)	Gender	Post stroke started at the facility (weeks)
B1	55	Male	32
B2	21	Female	78
B3	34	Male	11
B4	38	Male	30
B5	41	Female	58

### Disability

On admission into the programme, all stroke survivors had no finances to access private, intensive rehabilitation. They had all been to government facilities previously. Table 2 presents their baseline disability, goals and discharge outcomes.

**TABLE 2:** Disability manifestation, goals and discharge outcomes of the stroke survivors.

Stroke survivor	Disability manifestation (at baseline)	Goals	Discharge outcomes
B1	**Impairments:** Right hemiparesis, aphasia, dysphagia, dysarthria, cognitive impairments and right shoulder subluxation **Activity limitation and participation restrictions:** Wheelchair-bound, could not move from sit-to-stand and could not stand. Required assistance with transfers, mobility and all activities of daily living (ADLs) **Personal factors**: Male, mid-fifties, lived with a partner, enjoyed TV and socialising with family and friends **Contextual factors**: Unemployed and worked as a machine operator, enjoyed attending church	**Communication:** Communicate with improved speech and use augmentative and alternative communication (AAC) with prompting **ADLs:** Eat, drink, groom, attend to personal care transfer from one surface to another independently and ambulate with aid or support **Other:** Eat solid food	**Goals** **Communication:** Could communicate using the AAC with prompting **ADLs:** Could transfer independently, groom, care for self and ambulate with support **Other:** Could not eat normal food because of dysphagia at discharge **Outcome measures** **Function:** The FIM+FAM score increased from 108 at baseline to 130 at 6 months (discharge) **Quality of life:** The EQ-5D-5L scores reduced from 25 at baseline to 11 at 6 months (discharge) **Depressive symptoms:** Depressive symptoms score decreased from 13 at baseline to 8 at 6 months (discharge)
B2	**Impairments:** Right hemiparesis, Broca’s aphasia and apraxia, low mood, cognitive difficulties, poor balance and antalgic gait **Activity limitation and participation restrictions:** Partially wheelchair-bound. Could slowly walk short distances with a crutch **Personal factors:** Female, early 20s, university student, lived with family. Planned to return to school **Contextual factors:** Active in church, used to socialise but hardly went out because of the disability	**Communication:** Use the AAC to communicate and basic communication skills **Function:** Use the right hand to eat and write **ADLs:** Assist with cooking and cleaning, groom, caring for self and transferring from one surface to another independently **Return to work:** Study social work or a short course **Social participation:** Socialise with friends, attend church and shop Other: Read the Bible	**Goals** **Communication:** Able to communicate with AAC **Function:** Able to use right hand to eat and write **ADLs:** Independent with grooming, self-care, transferring from one surface to another and assisting with cooking in the home **Social participation:** Could socialise with friends and attend church/shop **Return to work:** The goal to return to school was not achievable during the intervention period **Other**: Able to read the Bible **Outcome measures** **Function:** The FIM+FAM score increased from 140 at baseline to 175 at 12 months (discharge) **Quality of life:** The EQ-5D-5L scores increased from 10 at baseline to 11 at 12 months (discharge) **Depressive symptoms:** Depressive symptoms score decreased from 12 at baseline to 10 at 12 months (discharge)
B3	**Impairments:** Left hemiparesis, dysarthria, left upper limb spasticity, fatigue, low mood and blunted affect, reduced insight and awareness, impaired attention and memory skills **Activity limitation and participation restrictions:** Mobilised with a crutch and required assistance to perform ADLs **Personal factors**: Male, mid-30s, moved in with mother after stroke and initially continued to receive income from work **Contextual factors**: Worked as a gas technician, enjoyed socialising and cooking, breadwinner for 3 children	**Communication:** Improve cognitive communication skills **Function:** Use the left upper limb for functional activities **Social skills:** Demonstrate verbal and non-verbal skills when interacting **ADLs:** Cook, ambulate indoors and outdoors, climb stairs and use public transportation **Return to work:** Return to work part-time and be evaluated for fitness to drive assessment	**Goals** **Communication:** Cognitive communication skills improved **Function:** Could use the left upper limb for functional activities **Social skills:** Able to use verbal and non-verbal skills when interacting **ADLs:** Could cook, ambulate indoors and outdoors, climb stairs and use public transportation **Return to work:** B3 was recommended to drive an automatic car. He returned to work shortly after discharge as a storeman **Outcome measures** **Function**: The FIM+FAM score increased from 170 at baseline to 202 at 12 months (discharge) **Quality of life:** The EQ-5D-5L scores reduced from 11 at baseline to 7 at 12 months (discharge) **Depressive symptoms:** Depressive symptoms score increased from 4 at baseline to 15 at discharge
B4	**Impairment:** Right hemiparesis, expressive aphasia, verbal apraxia and poor balance **Activity limitation and participation restrictions:** Could mobilise with support, could transfer slowly but mainly wheelchair-bound. Dependent for all ADLs **Personal factors:** Male, late-30s, employed (on disability leave), received income from work, lived with relative as unable to access own home living with a partner **Contextual factors:** Previously lived with a partner, had a supportive sibling, worked as a driver and enjoyed watching and playing soccer	**Communication:** To use the AAC system, establish ‘yes’ or ‘no’ communication and be able to say 5–10 words **Function:** Multitask with carrying objects **ADLs:** To perform grooming and personal care activities, transfer, ambulate in the house, walk-in public and use public transportation **Return to work**: To start a business	**Goals** **Communication:** Could use the AAC, say yes or no and say 5–10 words **ADLs:** Could clean, groom, shop, care for self, transfer and ambulate in the house and outside and use public transportation **Function:** Could multitask especially when carrying an object **Return to work:** B4 could not start a business during the intervention period **Outcome measures** **Function**: The FIM+FAM score increased from 152 at baseline to 182 at 12 months (discharge) **Quality of life**: The EQ-5D-5L scores reduced from 14 at baseline to 7 at 12 months (discharge) **Depressive symptoms**: Depressive symptoms score increased from 2 at baseline to 6 at 12 months (discharge)
B5	**Impairment:** Left hemiparesis, mild to moderate cognitive-communication difficulties, no active hand and finger movement **Activity limitation and participation restrictions:** Mobilised unaided with poor gait pattern at a gait speed of 0.72m/s **Personal factors**: Female, early 40s with 3 children, received partial income (on disability leave) **Contextual factors:** Married but did not live with partner. No other social support	**Social participation:** To increase confidence in community engagements **ADLs:** To prepare meals, hang clothes, clean, carry a cup and bucket of water, ambulate indoors and outdoors, climb stairs and use public transportation **Return to work:** To return to work as a cleaner	**Goals** **Social participation:** Confidence in community engagements was increased during the intervention period **ADLs:** Could prepare meals, hang clothes, clean, carry a cup and a bucket of water, ambulate indoors and outdoors, climb stairs and use public transportation **Return to work:** Could not return to work as a cleaner during the intervention period **Outcome measures** **Function**: The FIM+FAM score increased from 173 at baseline to 195 at 6 months (discharge) **Quality of life**: The EQ-5D-5L scores reduced from 15 at baseline to 11 at 6 months (discharge) **Depressive symptoms**: Depressive symptoms score decreased from 22 at baseline to 10 at 6 months (discharge)

FIM, functional independence; B, beneficiary; ADLs, activities of daily living; FIM, functional independence measure; FAM, functional assessment measure.

### Stroke survivor goals

As shown in Table 2, in general, goals set focussed on improving function, ADLs, social participation, communication and return to work. Most stroke survivors’ goals were achieved or partially achieved within the first 6 months of the programme. For the programme’s first 3 months, the goals were focussed mainly on function, social participation and ADLs. In the second and third quarters of the programme, the stroke survivors’ goals were geared towards returning to work. Although these return-to-work goals were mostly not achieved, the programme appeared to place them on a trajectory that introduced them and prepared them for future vocational opportunities by maximising their level of function to adapt to their current work interest or alternate work entirely. Table 3 outlines the specific goals and goals achieved over the period of the programme.

**TABLE 3:** Stroke survivors’ set and achieved goals.

Stroke survivor	Goal set	Goal achieved (months)			
		3	6	9	12
**B1**					
Communication	Communicate with improved speech	NA	-	-	-
	Use the AAC system with prompting	-	A	A	-
Function	Eat and drink	A	-	-	-
Social participation	-	-	-	-	-
IADL	-	-	-	-	-
BADL	Grooming	PA	A	A	-
	Personal care	PA	PA	A	-
	Transfer	A	-	-	-
	Ambulate with aid or support	PA	PA	A	-
Return to work	-	-	-	-	-
Personal	Eat normal diet	NA	-	-	-
**B2**					
Function	Use the right hand to eat and write	NA†	-	-	-
Social participation	Socialise with friends	NA	PA	PA	A
	Attend church and shop	NA	PA	PA	A
IADL	Assist with cooking	-	PA	PA	A
	Assist with cleaning	NA	PA	-	-
BADL	Grooming	PA	PA	PA	A
	Personal care	-	PA	PA	A
	Transfer	-	-	A	-
Communication	Use the AAC app to communicate	-	-	PA	A
	Basic communication skills	-	-	-	-
Return to work	Study social work or a short course	NA†	-	-	-
Personal	Read the Bible	PA	PA	PA	A
**B3**					
Communication	Improve cognitive-communication skills	A	-	-	-
Function	Use left UL	A	-	-	-
Social	Demonstrate verbal and non-verbal skills when interacting	-	-	PA	A
IADL	Prepare meal	-	-	A	-
	Time management		-	-	-
BADL	Ambulate indoors and outdoors	A	-	-	-
	Climb stairs	A	-	-	-
	Use public transportation	-	-	A	-
Coping skills	-	A	-	-	-
Return to work	Return to work part time	-	-	NA	-
	Fitness to drive assessment	-	-	NA	PA
**B4**					
Communication	Use AAC	NA	NA	NA	A
	Establish yes or no	A	-	-	-
	Say 5–10 words	-	-	PA	-
Function	Multitasking with carrying an object	-	-	-	A
Social	Shop	-	-	A	-
IADL	Cleaning	-	A	-	-
BADL	Grooming	-	A	-	-
	Personal care	A	-	-	-
	Transfer	-	-	-	-
	Ambulate in the house	A	-	-	-
	Walk in public	-	A	-	-
	Use public transportation	NA	NA	NA	A
Return to work	Start a business	-	-	-	NA
**B5**					
Communication	-	-	-	-	-
Function	-	-	-	-	-
Social	Increase confidence in community engagements	-	A	-	-
IADL	Stabilise food during meal prep	-	A	-	-
	Hang clothes	PA	A	-	-
	Clean	PA	A	-	-
	Carry a bucket of water	-	A	-	-
BADL	-	-	-	-	-
	Use public transportation	A	-	-	-
	Ambulate – change direction indoors and outdoors	A	-	-	-
	Carry a cup of tea independently	-	A	-	-
	Climb stairs	-	A	-	-
Return to work	Return to work as a cleaner	-	NA	-	-

A, achieved; PA, partially achieved; NA, not achieved; AAC, augmentative and alternative communication; IADL, instrumental activities of daily living; BADL, basic activities of daily living; UL, Upper Limb.

† , terminated.

**FIGURE 2:**
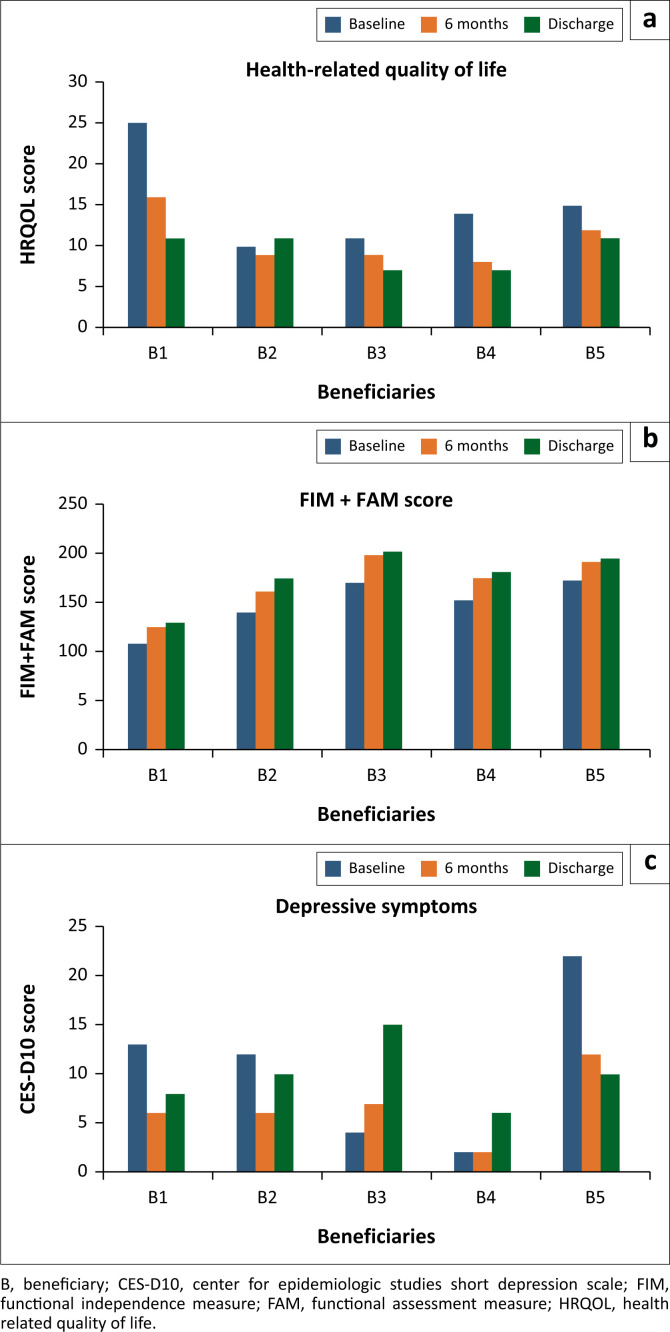
Baseline, 6 months and discharge outcome measures of functional independence, health-related quality of life and depressive symptoms of the stroke survivors.

Figure 2 shows the CES-D10, FIM+FAM and EQ-5D-5L scores for all participants at baseline, 6 months and at discharge demonstrated improvements for all three outcomes. At baseline, three stroke survivors (B1, B2 and B5) had CES-D10 ≥ 10, and for these three, the CES-10 score decreased at 6 months and discharge, compared to the baseline, indicating a reduction in depressive symptoms. However, it is interesting to note that B3 presented with an increased score over the course of the programme, which indicated an increase in clinically significant depressive symptoms despite their improved FIM and quality of life (Figure 2). The FIM+FAM score compared to the baseline was higher in all the beneficiaries at 6 months and upon discharge, demonstrating improved FIM. At baseline, the FIM+FAM scores ranged from 108 to 173, and at discharge, they ranged from 130 to 202 (Figure 2). The health-related quality of life score decreased in all the beneficiaries at 6 months and discharge compared to the baseline, demonstrating improved quality of life. At baseline, the EQ-5D-5L scores ranged from 10 to 25, and at discharge, the scores ranged from 7 to 11 (Figure 2).

## Discussion

The BBJ project, using a community-based, interdisciplinary approach, was conceptualised to restore FIM, social participation and quality of life to stroke survivors. Between 2019 and 2021, five stroke survivors consecutively participated in the programme. The outcomes showed that via engagement with this programme, these five stroke survivors achieved most of their goals regarding FIM, cognitive functioning, social participation and improved daily living activities.

Functional independence and quality of life had improved at discharge in all stroke survivors. This is not surprising as evidence has shown that rehabilitation interventions improve quality of life, cognitive functioning and FIM post-stroke (Gao et al. 2024). However, the evaluation of depressive symptoms at baseline and discharge did not show similar outcomes. Only one stroke survivor (B1) reported a clinically significant reduction in depressive symptoms (from CESD10 ≥ 10 to CESD10 < 10). An interesting finding was the increase in clinically significant depressive symptoms (from CESD10 < 10 to CESD10 ≥ 10) in B3 at discharge compared to baseline. The presence and persistence of depressive symptoms and anxiety post-stroke are common whether or not the stroke survivors have access to rehabilitation programmes (Hartley, Burger & Inglis-Jassiem 2022; Mahadevan et al. 2021), and it has been reported that depressive symptoms and anxiety are more common in younger adults compared to older people (> 50 years) (Kapoor et al. 2019). In our study, the only stroke survivor (B1) who had a clinically significant reduction in depressive symptoms was also the oldest (mid-fifties), while the others were within the younger adult age group.

Some dynamics of living with stroke as a younger person are different from those of an older adult, as the impact of stroke on the life of a younger person is sometimes invisible and could impact other outcomes (Lawrence 2010). Disorientation, life (social and psychological) disruption and change in roles and relationships are three main themes identified in a qualitative systematic review of experiences of young adults post-stroke (Lawrence 2010). Other themes identified from the literature include – loss of valued activities (recreation and employment) (Kuluski et al. 2014) and loss of pre-stroke life construct and relationships (Shipley et al. 2018). Navigating life as a young adult post-stroke can be challenging, and the burden could increase in a community with a high potential to stigmatise people living with disability (Trani et al. 2020). An understanding of the contextualised burden of living with stroke as a young person in a low-income community in South Africa could help in mapping out potential strategies to mitigate the challenges associated with living with stroke as a young person, which could improve psychological outcomes.

No stroke survivor fully achieved the return-to-work goal at discharge. South Africa is grappling with high unemployment rates (Stats SA 2023), which presents a challenge for people living with disabilities to get jobs as available work opportunities are dwindling. The rate of return to work among stroke survivors ranges from 12%–64% in various populations (Baldwin & Brusco 2011; Olaoye, Soeker & Anthea 2021). In a randomised controlled trial conducted in South Africa, after a workplace intervention programme, 60% (*n* = 24) of stroke survivors in the intervention group returned to work compared to 20% (*n* = 8) in the control group. In this case series, the stroke survivors who did not return to work, unwillingness by old employers to employ them, as well as cognitive, psychological and functional challenges, were the primary reasons (Ntsiea et al. 2015). All the stroke survivors of our programme found that returning to their previous jobs was challenging because of their disabilities, and therefore, they would require skill adaptation to seek employment.

Furthermore, the individual and community understanding of the dynamics of disability and ability was a challenge. There was a sense of incapacitation associated with living with a disability at the individual level and a community approach to people with disabilities, which hindered their ability and desire to return to work. Despite participants’ willingness to return to work, their family members sometimes did not encourage it, and the wider community questioned their cognitive and physical capacity to work. Additionally, previous employers are not willing to accommodate people with disabilities. A study conducted in a similar community in Soweto as our study showed that there is stigmatisation of people living with disabilities which can be seen in the higher levels of unemployment, which increases the risk of depression and low self-esteem (Trani et al. 2020). In people living with disabilities, being unemployed and having no education increase the risk of depression (Trani et al. 2020). Trani et al. (2020) recommend community engagement with people living with disabilities while providing psychosocial support and enacting anti-stigma policies.

## Recommendations

The ongoing promotion and facilitation of independence of the stroke survivors as they progress through their rehabilitation journey needs to be reinforced from admission and not just as FIM goals are achieved. As such, it is recommended to have follow-up sessions and check-ins post-discharge. In addition, all stroke survivors are encouraged to continue the support groups post-discharge from the formal programme. In future, meetings with the stroke survivor beneficiary manager and rehabilitation team should include the stroke survivor to ensure that the process is as person-centred as possible. An important learning point of our study was the stroke survivors’ return-to-work journey and the factors that negatively influenced their return to work. Return-to-work options should be considered by therapists and stroke survivors on admission and not only towards the end of the programme as this would allow more time to consider re-employment and reskilling opportunities before discharge. Partnering with business-oriented organisations that could assist with self-employment and business support would better support stroke survivors’ return-to-work goals.

## Conclusion

The individualised PSWI BBJ rehabilitation programme, which uses public–private partnerships to rehabilitate people with disabilities, has shown positive functional outcomes in an economically marginalised community. However, to further strengthen the programme, partnerships with business-oriented organisations to improve the return-to-work outcome would benefit the stroke survivors’ quality of life.
